# Prognostic Impact and Risk Factors of Infections in Patients with Chronic Lymphocytic Leukemia Treated with Ibrutinib

**DOI:** 10.3390/cancers13133240

**Published:** 2021-06-29

**Authors:** Francesca Romana Mauro, Diana Giannarelli, Andrea Visentin, Gianluigi Reda, Paolo Sportoletti, Anna Maria Frustaci, Annalisa Chiarenza, Stefania Ciolli, Candida Vitale, Luca Laurenti, Lorenzo De Paoli, Roberta Murru, Massimo Gentile, Gian Matteo Rigolin, Luciano Levato, Annamaria Giordano, Giovanni Del Poeta, Caterina Stelitano, Claudia Ielo, Alessandro Noto, Valerio Guarente, Stefano Molica, Marta Coscia, Alessandra Tedeschi, Gianluca Gaidano, Antonio Cuneo, Robin Foà, Maurizio Martelli, Corrado Girmenia, Giuseppe Gentile, Livio Trentin

**Affiliations:** 1 Hematology, Department of Translational and Precision Medicine, “Sapienza” University, 00161 Rome, Italy; claudia.ielo@uniroma1.it (C.I.); rfoa@bce.uniroma1.it (R.F.); martelli@bce.uniroma1.it (M.M.); girmenia@bce.uniroma1.it (C.G.); gentile@bce.uniroma1.it (G.G.); 2 Biostatistic Unit, Regina Elena National Cancer Institute, IRCCS, 00144 Rome, Italy; diana.giannarelli@ifo.gov.it; 3 Hematology and Clinical Immunology Unit, Department of Medicine, University of Padua, 35121 Padua, Italy; andrea.visentin@aopd.veneto.it (A.V.); livio.trentin@unipd.it (L.T.); 4 Fondazione IRCCS Cà Granda Ospedale Maggiore Policlinico, 20122 Milan, Italy; gianluigi.reda@policlinico.mi.it (G.R.); alessandro.noto@policlinico.mi.it (A.N.); 5 Institute of Hematology-Centro di Ricerca Emato-Oncologica (CREO), Department of Medicine, University of Perugia, 06129 Perugia, Italy; paolo.sportoletti@unipg.it (P.S.); valerio.guarente@studenti.unipg.it (V.G.); 6 Deptartment of Hematology, Niguarda Cancer Center, ASST Grande Ospedale Metropolitano Niguarda, 20162 Milano, Italy; annamaria.frustaci@ospedaleniguarda.it (A.M.F.); alessandra.tedeschi@ospedaleniguarda.it (A.T.); 7 Division of Hematology, Ferrarotto Hospital, 95123 Catania, Italy; annalisa.chiarenza@gmail.com; 8 Hematology Unit, Careggi Hospital, 50139 Firenze, Italy; ciollis@aou.careggi.toscana.it; 9 Division of Hematology, A.O.U. Città della Salute e della Scienza di Torino and Department of Molecular Biotechnology and Health Sciences, University of Turin, 10126 Turin, Italy; candida.vitale@unito.it (C.V.); marta.coscia@unito.it (M.C.); 10 Institute of Haematology, Fondazione Policlinico Universitario A. Gemelli IRCCS, 00168 Rome, Italy; Luca.Laurenti@unicatt.it; 11 Division of Hematology, Department of Translational Medicine, University of Eastern Piedmont, 28100 Novara, Italy; lorenzo.depaoli@med.uniupo.it (L.D.P.); gianluca.gaidano@med.uniupo.it (G.G.); 12 Haematology and Stem Cell Transplantation Unit, Ospedale Oncologico A. Businco, AO Brotzu, 09134 Cagliari, Italy; roberta.murru@tiscali.it; 13 Hematology Unit, Hematology and Oncology Department, 87100 Cosenza, Italy; massim.gentile@tiscali.it; 14 Hematology, Department of Medical Sciences, St. Anna University Hospital, 44124 Ferrara, Italy; rglgmt@unife.it (G.M.R.); cut@unife.it (A.C.); 15 Haematology Unit, A. Pugliese Hospital, Azienda Ospedaliera Pugliese Ciaccio, 88100 Catanzaro, Italy; leluc13@alice.it (L.L.); smolica@libero.it (S.M.); 16 Department of Emergency and Organ Transplantation (D.E.T.O.), Hematology Section, University of Bari, 70124 Bari, Italy; annamaria.giordano1977@gmail.com; 17 Hematology, Department of Biomedicine and Prevention, University Tor Vergata, 00133 Rome, Italy; g.delpoeta@tin.it; 18 Division of Hematology, Azienda Ospedaliera Bianchi-Melacrino-Morelli, 89124 Reggio Calabria, Italy; caterinastelitano27@gmail.com

**Keywords:** chronic lymphocytic leukemia, ibrutinib, infection, prognosis

## Abstract

**Simple Summary:**

Ibrutinib demonstrated superior efficacy compared to chemoimmunotherapy in patients with chronic lymphocytic leukemia. However, adverse events are a frequent reason for treatment discontinuation. This study was aimed to evaluate the incidence, risk factors, and prognostic impact of infections in a large series of patients with chronic lymphocytic leukemia who received an ibrutinib-based therapy. Approximately one-third of patients developed pneumonia or a severe infection with an overall rate of 15.3% infections per 100 person-year. Patients who experienced a severe infection in the year before starting ibrutinib, those with chronic obstructive pulmonary disease, and those heavily pretreated showed greater vulnerability to infection. A scoring system based on these factors identified patients with a two- to threefold increase in the rate of infections. Infections showed an unfavorable impact in terms of treatment discontinuation and inferior survival. Our results demonstrate that infections are a relevant reason for treatment failure in patients treated with ibrutinib.

**Abstract:**

Ibrutinib represents extraordinary progress in the treatment of chronic lymphocytic leukemia (CLL). However, treatment-related adverse events limit the benefit of this agent. This observational, multicenter study focused on the incidence, risk factors, and prognostic impact of infections in 494 patients with CLL treated with an ibrutinib-based treatment. Ibrutinib was given to 89 (18%) previously untreated patients (combined with rituximab, 24) and 405 (82%) relapsed/refractory patients. Pneumonia (PN), grade ≥3 non-opportunistic infections (NOI), and opportunistic infections (OI) were recorded in 32% of patients with an overall incidence rate per 100 person-year of 15.3% (PN, 10%; NOI, 3.3%; OI, 2%). Infections were the reason for the permanent discontinuation of ibrutinib in 9% of patients. Patients who experienced pneumonia or a severe infection showed a significantly inferior survival than those who were infection-free (*p* < 0.0001). A scoring system based on the three factors associated with a significant and independent impact on infections—PN or severe infection in the year before starting ibrutinib, chronic obstructive pulmonary disease, ≥2 prior treatments—identified patients with a two- to threefold increase in the rate of infections. In conclusion, the results of this study highlight the adverse impact of infectious events on the outcomes of CLL patients treated with ibrutinib.

## 1. Introduction

Chronic lymphocytic leukemia (CLL) is the most common leukemia in the adult population. According to the National Cancer Institute’s Surveillance, Epidemiology, and End Results Program (SEER) [1], the estimated number of new cases of CLL in the United States in 2021 is 21,250. The incidence rapidly increases with increasing age, and the median age at diagnosis is about 70. The CLL-IPI combines genetic, biochemical, and clinical parameters into a prognostic model, discriminating four prognostic subgroups with 5-year survival probabilities ranging from 93% to 23% [2].

Several randomized trials have demonstrated the superiority of the B-cell receptor pathway inhibitor ibrutinib over chemotherapy in patients with chronic lymphocytic leukemia (CLL). [3,4,5,6]. The remarkable activity and the favorable tolerability profile have favored the widespread use of ibrutinib for the upfront treatment of patients with CLL. However, treatment discontinuation due to specific off-target toxicities of this agent, such as bleeding events and atrial fibrillation, is not negligible, although some treatment-related adverse events are now better managed [7,8,9,10,11,12,13,14,15].

It is well known that defects in humoral and cellular immunity due to CLL itself and chemoimmunotherapy result in an increased risk of infections that are a significant cause of morbidity and mortality in CLL patients [16]. Recently, an increased rate of infections has also been described in patients treated with ibrutinib, particularly in R/R patients. As a lower risk of infectious events was expected with targeted agents than chemoimmunotherapy, several reports have focused on infections in patients treated with ibrutinib [16,17,18,19,20,21,22,23,24,25,26,27,28,29,30,31,32,33,34]. Although several inhibitory effects exerted by ibrutinib on immune effector cells have been described to explain the increased fragility to infections (particularly fungal infections [35,36,37,38,39,40,41,42]), little is known about the prognostic impact of infections in patients treated with ibrutinib. Moreover, the role of clinical and biological factors in favoring infections in patients treated with ibrutinib should be better defined.

This retrospective, multicenter study was carried out to define the incidence of pneumonia and severe infections in a large series of patients with CLL treated with ibrutinib. This study was also aimed at investigating risk factors and the impact of infections on treatment discontinuation and survival of patients receiving ibrutinib.

## 2. Materials and Methods

This study analyzed retrospectively the characteristics of 494 patients with CLL treated with ibrutinib at 16 Italian institutions, 12 academic centers, and four hospitals.

Inclusion criteria were CLL diagnosis according to the iwCLL criteria [43] and front-line or advanced-line treatment with ibrutinib-based therapy having started between February 2013 and February 2019. Exclusion criteria included known transformation from CLL to an aggressive lymphoma (i.e., Richter transformation) and clinically significant comorbidities potentially interfering with the regular administration of treatment.

Data of patients were collected from medical records by the referring physician and included demographics, comorbidities, infectious events within 12 months before starting ibrutinib, prior treatment, serum IgG level, antimicrobial prophylaxis, concomitant use of steroids for more than four weeks, the IGHV and*TP*53 mutation status, and the FISH profile.

Three types of infections were considered: pneumonia (PN), grade ≥ 3 non-opportunistic infections (NOIs), and opportunistic infections (OIs). When available, the etiologic agent was recorded. Infections of non-bacterial etiology with lung involvement were classified as NOI or OI, according to the identified agent. The severity of infections was graded according to the Common Terminology Criteria for Adverse Events, version 4.0.

The primary endpoint of this study was the incidence of PN, NOIs, and OIs in patients receiving ibrutinib. The secondary endpoints were the impact of infections on treatment discontinuation, survival, and factors associated with an increased rate of infectious events.

The database was locked on March 30, 2020, for analysis. The person-year duration of ibrutinib exposure was defined for each patient as the time from the start of ibrutinib to the last taken dose, last follow-up, or death. The person-year incidence rate of infections was defined as the number of infectious events observed during treatment divided by the total person-years of ibrutinib exposure. Survival was calculated from the start of treatment to the infectious event, disease progression, Richter syndrome, or the last follow-up or death. Survival curves were calculated according to the Kaplan and Meier method and differences in survival using the log-rank test in univariate analysis and the Cox regression model in multivariate analysis, after the assessment of the proportionality of hazards. Variables associated with a significant and independent increase in the infection rate were selected using a stepwise forward method, based on Wald statistics [44]. A scoring system to assess the risk of infections was developed by assigning to each significant variable in the final model a weighted point based on its hazard ratio, proportional to the regression β coefficient. The bootstrap method was used to validate the model. Prediction performance has been evaluated by the area under the curve (AUC) of the receiver operating characteristic (ROC) curve. Confidence intervals (CIs) have been calculated at the 95% level. All statistical tests were two-sided. A p-value of less than 0.05 has been considered significant. All analyses have been performed in SPSS v26.

This observational study was conducted in accordance with the Declaration of Helsinki and approved by the institutional review board and ethical committee.

## 3. Results

### 3.1. Clinical Characteristics of Patients

The baseline characteristics of the 494 CLL patients included in this study are detailed in Table 1. The median time on ibrutinib was 35 months (range, 4–85 months) and the median duration of the exposure to ibrutinib per patient was 2.6 years. The median age of patients was 69 years (range, 32–92 years). A CIRS >6 was observed in 24% of patients and creatinine clearance <70 mL/min in 37.4%. A chronic obstructive pulmonary disease was recorded in 16% of patients, and 24% had a grade ≥3 severe infection or a pneumonia event in the year before starting ibrutinib. Twenty-one percent of patients showed IgG levels ≤ 400 mg/dl and were on immunoglobulin support and 8% had a baseline granulocyte count <1 × 109/L. Rai stage III-IV was present in 53% of patients, unmutated IGHV status in 73.5%, and *TP*53 disruption (del17p, and or *TP*53 mutation) in 55.2%.

Eighty-nine (18%) of the patients received front-line therapy with ibrutinib, 420 mg once daily given continuously and 24 received also rituximab, 375 mg/sqm, weekly on day 1 of month 1 and then on day 1 of months 2–6. After a median number of one prior treatment (range, 1–9), 405 (82%) relapsed/refractory patients received ibrutinib single agent, 420 mg once daily given continuously. Concomitant steroids have been administered to 17% of patients.

Infection prophylaxis did not follow common guidelines but rather the guidelines of each institution. Seventy-four percent of patients were on *Pneumocystis jirovecii* (PJ) prophylaxis with trimethoprim-sulfamethoxazole (TMP-SMX), and 53 (8%) patients with a known risk of hepatitis B reactivation (HBc+ and, or HBsAg+) received lamivudine.

### 3.2. Incidence of Infections

One hundred and fifty-six (32%) patients experienced at least one infectious event and 35 (7%) had an additional infection. The total number of infections we recorded was 193 with an overall incidence rate of 15.3% infections per 100 person-year. Pneumonia was the most common infection with an incidence per 100-person per year of 10%, while it was 3.3% for grade ≥3 non-opportunistic infections, and 2.0% for opportunistic infections. The median time from the start of ibrutinib and the onset of infections was six months, seven for pneumonia events, nine for grade ≥3 non-opportunistic infections, and three for opportunistic infections (Table 2).

### 3.3. Type of Infections

The incidence and type of infections are described in detail in Table S1.

One hundred (20%) patients experienced at least a pneumonia event, 32 (6.5%) a grade ≥3 non-opportunistic infection, and 24 (5%) a grade ≥3 opportunistic infection. Among the 32 patients who developed a grade ≥3 non-opportunistic infection, 7 viral infections were recorded. HBV reactivation was described in three patients in whom the serological profile for HBV had not been evaluated at baseline while none of the 41 patients on HBV prophylaxis due to a known risk for HBV reactivation developed an HBV reactivation. Symptomatic COVID-19 pneumonia was diagnosed in two young patients with hypogammaglobulinemia and was fatal in one. Fungal infections were the most frequent type of opportunistic infection (14/24; 58%), and, in turn, *Aspergillus* infection was the most frequent type of fungal infection (11/14; 79%). *Aspergillus* infection was associated with SNC involvement in three cases and was the direct cause of death for 6/11patients. Other opportunistic infections included C*ryptococcus* infection in two patients, CMV infection in three, disseminated HVZ infection in five. *Pneumocystis jirovecii* pneumonia (PJP) was diagnosed in a previously treated patient who received trimethoprim-sulfamethoxazole irregularly. This accounts for 0.8% (1/129) of PJP cases among patients who did not receive appropriate prophylaxis. Mycobacterium infection was described in two asymptomatic patients who underwent the biopsy of an isolated lung nodule. A fatal, progressive multifocal leukoencephalopathy was recorded in a 79-year-old man with advanced disease treated with ibrutinib and rituximab.

### 3.4. Outcomes of Patients Who Developed Infections

Forty-three (9%) patients discontinued ibrutinib permanently due to an infection. Treatment discontinuation due to infection was an early event observed in 20 (4%) patients in the first year of treatment, in 10 (2%) in the second year, and 13 (3%) thereafter.

The other most frequent reasons leading to permanent ibrutinib discontinuation were disease progression in 16% of patients, Richter syndrome (RS) in 5%, atrial fibrillation in 5%, and second malignancies in 4% (Table S2).

Infections were the direct cause of death for 29/494 (6%) patients (Table 2). Other causes of death were disease progression (6%) and Richter syndrome (5%) (Table S3).

**Figure 1:**
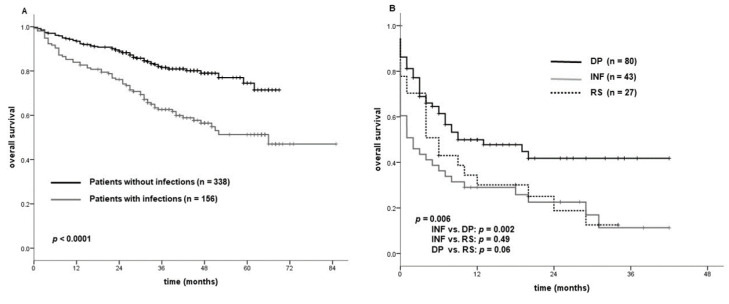
(**A**) Survival in patients with and without an infectious event. (**B**). Survival probability from the time of disease progression (DP), infection (INF), Richter syndrome (RS).

Patients who experienced pneumonia or a severe infection showed a significantly inferior survival than those who were infection-free (36-month OS: 82% vs. 63%; *p* < 0.0001; Figure 1A). The survival probability of patients who discontinued ibrutinib due to an infection or Richter syndrome was significantly inferior to that of those who discontinued treatment due to disease progression (median survival: 2 vs. 6 vs. 9 months; *p* = 0.006) (Figure 1B).

### 3.5. Impact of Baseline Characteristics of Patients on Infections

The incidence rates per 100 person-year of infections according to patients’ clinical and biologic characteristics are summarized in Table 3. Baseline factors associated with a significantly higher rate of infections were chronic obstructive pulmonary disease (*p* <0.0001), pneumonia or grade ≥3 infection in the year before starting ibrutinib (*p* < 0.0001), IgG levels ≤400 mg/dl (*p* = 0.002), neutrophil count <1 × 109/L (*p* = 0.001), Rai stage III-IV (*p* < 0.0001), prior treatment (0 vs. ≥1, *p* = 0.004; 1 vs. ≥ 2; *p* < 0.0001), and the addition of steroids to ibrutinib (*p* = 0.002) (Table 3). The addition of rituximab to front-line therapy with ibrutinib, the IGHV mutational status, and the presence of *TP*53 aberrations did not reveal any impact on the infection rate.

In multivariate analysis, three factors maintained significance: a pneumonia event or a severe infection in the year before starting ibrutinib (HR, 2.69 (95% CI, 1.90–3.76)), the presence of a chronic obstructive pulmonary disease (HR, 1.52 (95% CI, 1.03–2.25)), two or more prior treatments (HR, 1.63 (95% CI, 1.17–2.28)) (Table 4).

To predict the risk of infections we designed a scoring system based on the HR values of factors with a significant and independent impact on infections. Two points were assigned for the presence of a pneumonia event or a severe infection in the year before starting ibrutinib, and one for chronic obstructive pulmonary disease, and two or more prior treatments. The sum of these scores showed an AUC of 0.65 (95% CI: 0.60–0.71). Three subgroups of patients were identified according to the score. The low-risk group (score, 0–1 points) identified 352 (71.2%) patients of whom 81 (23.0%) experienced an infectious event. The intermediate-risk group (score, 2 points) included 67 (13.6%) patients of whom 28 (41.8%) experienced an infection. Finally, the high-risk group (score, ≥3 points) identified 75 (15.2%) patients of whom 47 (62.7%) developed an infection. (Table 5).

## 4. Discussion

Infections are a well-known cause of morbidity and mortality in CLL patients treated with chemotherapy. This study was carried out to evaluate the prognostic impact of pneumonia and severe infections in patients who receive ibrutinib.

Overall, with a median follow-up of 35 months, about a third of patients experienced at least one infectious event with an overall rate of 15.3% infections per 100 person-year. Given the large proportion of unselected and previously treated patients included in this study, the high infection-related morbidity was not unexpected as compared to that described in controlled trials [3,4,5,6,9,10,45,46]. Previous chemotherapy treatment revealed a significant impact in promoting greater fragility to infections in patients receiving ibrutinib.

As observed in other studies, infections were an early event that occurred after a median time of 7 months from starting ibrutinib [10,46]. This observation suggests careful monitoring of patients during the first months of treatment with ibrutinib. Pneumonia was recorded in 20% of patients with an incidence rate per 100 person-years of 10%. Variable rates of pneumonia events, ranging between 10% and 28%, have been reported and this could be related to the different characteristics and follow-up of patients included in other studies [3,4,5,6,9,32,34,46].

Two young, R/R patients with hypogammaglobulinemia developed symptomatic COVID-19 infection, discontinued treatment, and one did not survive the infection. The small number of patients with COVID-19 disease we recorded was due to the locking of the database for analysis before the pandemic became widespread in our country. At present, there is no agreement on the impact of ibrutinib on the outcomes of CLL patients who developed COVID-19 disease. Some studies report a lower number of COVID-19 infections than expected and more favorable outcomes in patients treated with ibrutinib [47,48,49,50,51]. However, the same favorable impact of ibrutinib has not been confirmed in a multicenter study [52].

The rate of opportunistic infections per 100 person-year recorded in this study, 2%, is in line with that reported by Rogers et al., 1.9% [23]. Consistent with other reports, the most common fungal infection was due to the *Aspergillus fumigatus* [24,25,26,27,28]. It has been argued that the inhibitory effects of ibrutinib on the activity of T-cells, natural killer cells, and macrophages may play a role in the development of these infections [36,37,38,39,40,41]. Given the relatively low incidence, routine prophylaxis of fungal infections has not been recommended. Similarly, the low incidence of PJP in this study, as well as in other studies [28,29], makes one also question the need for specific prophylaxis in patients receiving ibrutinib. However, the presence of an opportunistic infection should be kept in mind, in patients who develop clinical signs of a severe infection.

Treatment discontinuations due to infections were more frequently observed than those due to other treatment-related adverse events, such as atrial fibrillation and bleeding events. Patients who experienced pneumonia or a severe infection showed a significantly inferior survival than those who were infection-free. Moreover, patients who developed pneumonia or a severe infection showed a significantly inferior survival than those who progressed. These findings underline the unfavorable impact of infections and the need to identify factors associated with a higher risk of infectious events. Three baseline factors revealed a significant and independent impact on the infection risk. The first one, an infectious event in the year preceding the start of ibrutinib, emphasizes the impact of a pre-existing immunodeficiency in predisposing further infections. The second, the presence of a concomitant chronic obstructive pulmonary disease, is a well-known risk factor for respiratory infections. Finally, the third factor, heavy pretreatment, is also a well-known clinical condition associated with an increased risk of infections. In this study, pretreated patients showed double the incidence of infections compared to treatment-naive patients. Earlier use of ibrutinib can result in a lower rate of infectious events.

Concerns about infectious events led several scientific groups to recommend more attention to the infection risk of patients treated with BTK inhibitors and other targeted molecules [12,13,14,15,53]. Other BTK inhibitors, acalabrutinib, zanubrutinib, show a more favorable safety profile [54,55]. Interestingly, in contrast to ibrutinib, tirabrutinib does not show inhibition of T lymphocyte function and this could result in less susceptibility to infections [56].

The scoring system we designed requires an external validation cohort to confirm its predictive value. However, it identified about 30% of patients with a two- to threefold increase in the rate of infectious events. These more vulnerable patients could benefit from measures to prevent and mitigate the harmful impact of infections.

## 5. Conclusions

The results of this study show that infections are still a critical issue in the treatment management of CLL patients who receive ibrutinib and highlight the adverse impact of infectious events on treatment discontinuation and survival.

## Figures and Tables

**Table 1:** Baseline characteristics of CLL patients treated with ibrutinib ± rituximab.

	*n* (%)
Number of patients	494 (79.7)
Median duration of exposure to ibrutinib, months (range)	35 (4–85)
Gender male Gender female	338 (68.4) 156(31.6)
Median age, years (range)	69 (32–92)
Median time from CLL diagnosis, years (range)	6 (3–34)
Median CIRS, (range)	4 (0–16)
Patients with CIRS > 6	185 (37.4)
Patients with CrCl < 70 mL/min	167 (33.8)
Patients with COPD	78 (15.8)
Smokers	123 (24.9)
Patients with diabetes	78 (15.8)
Pneumonia or grade ≥3 infections within 1 year before starting ibrutinib	118 (23.9)
Median IgG levels, mg/dL (range)	625 (82–5220)
Patients with IgG levels ≤ 400 mg/dL on Ig support	99/472 (21.0)
Median Hb × 10^9^/L (range)	12.0 (3.8–17.4)
Median neutrophil count × 10^9^/L (range)	3.2 (0.11–9.9)
Patients with neutrophil count < 1 × 10^9^/L	39 (8)
Median lymphocyte count × 10^9^/L (range)	36.7 (0.5–99.1)
Rai stage III-IV	260 (52.6)
Del17p and/or *TP*53 mutation	228/413 (55.2)
Unmutated IGHV	319/434 (73.5)
Mutated IGHV	115/434 (26.5)
CD38 ≥ 30%	130/335 (38.8)
Untreated patients ^(1)^	89 (18.0)
Previously treated patients	405 (82)
Median number of prior treatments (range)	1 (0–9)
Prior treatments = 1	169 (34.2)
Prior treatments = 2	101 (20.4)
Prior treatments > 2	135 (27.4)
Patients on TMP-SMX prophylaxis	365 (73.9)
Patients on HBV prophylaxis	41 (8.3)
Patients on fungal prophylaxis	1 (0.2)
Patients on concomitant steroids	83 (16.8)

Abbreviations: CIRS, Cumulative Illness Rating Scale; CrCL, creatinine clearance; COPD, chronic obstructive pulmonary disease; TMP-SMX, trimethoprim-sulfamethoxazole; IGHV, immunoglobulin heavy chain variable region genes; HBV, hepatitis B virus. ^(1)^ Ibrutinib single agent, 65 patients; ibrutinib+rituximab, 24 patients.

**Table 2:** Incidence and outcomes of pneumonia and grade ≥3 infectious events in patients treated with ibrutinib ± rituximab.

	*n* = 494 (%)
Ibrutinib exposure, person-years	1264
Median exposure to ibrutinib per patient, years	2.6
**Patients with at least pneumonia or grade ≥3 infection events**	156 (32)
Total pneumonia or grade ≥3 infection events	**193**
IR per 100 person-years	15.3
Median time to onset of the first infection, months (range) [IQR]	6 (0–54) (2–13)
Number of patients with pneumonia or grade ≥3 infections who discontinued ibrutinib	43 (9)
Number of patients with fatal pneumonia or grade ≥3 infections	29 (6)
**Patients with pneumonia**	
Number of patients with at least one pneumonia event	100 (20)
Total pneumonia events	126
IR of pneumonia events per 100 person-years	10.0
Median time to pneumonia, months (range) [IQR]	7 (0–51) [3,4,5,6,7,8,9,10,11,12]
Number of patients with pneumonia who discontinued ibrutinib	19 (4)
Number of patients with a fatal pneumonia	17(3.5)
**Patients with grade ≥3 non-opportunistic infections**	
Number of patients with at least a grade ≥3 non-opportunistic infection	32 (6.5)
Total grade ≥3 non-opportunistic infections	42
IR of grade ≥3 non-opportunistic infections per 100 person-years	3.3
Median time to grade ≥3 non-opportunistic infections, months, (range) [IQR]	9 (1–48) [5,6,7,8,9,10,11,12,13,14,15,16,17,18,19,20,21,22]
Number of patients with a grade ≥3 non-opportunistic infections who discontinued ibrutinib	10 (2)
Number of patients with a fatal grade ≥3 non-opportunistic infections	3 (0.6)
**Patients with grade ≥3 opportunistic infections**	
Number of patients with at least a grade ≥3 opportunistic infections	24 (5)
Total grade ≥3 opportunistic infections	25
IR of grade ≥3 opportunistic infections per 100 person-years	2.0
Median time to grade ≥3 opportunistic infections, months, (range) [IQR]	3 (0–54) [1,2,3,4,5,6,7,8,9,10]
Number of patients with a grade ≥3 opportunistic infection who discontinued ibrutinib	14 (3)
Number of patients with a fatal grade ≥3 opportunistic infection	9 (2)

Abbreviations: IR, incidence rate; IQR, interquartile range.

**Table 3:** Incidence rate per 100 person-years of pneumonia or grade ≥3 infections according to the clinical and biologic characteristics of patients treated with ibrutinib ± rituximab.

	Number of Patients *n* = 494	Number of Infections *n* = 193 (IR per 100 Person-Years = 15.3)	*p* Value
Gender M Gender F	338 156	127 (14.6) 66 (16.7)	0.38
Age ≥ 70 years	228	83 (14.7)	0.65
Age < 70 years	266	110 (15.7)	
CIRS ≥ 6	185	86 (17.8)	0.07
CIRS < 6	309	107 (13.7)	
CrCl ≥ 70 mL/min	327	124 (14.4)	0.23
CrCl < 70 mL/min	167	69 (17.2)	
Smokers	123	42 (14.0)	0.53
Non-smokers	371	151 (15.7)	
Patients with diabetes	78	32 (17.2)	0.46
Patients without diabetes	416	161 (14.9)	
Patients with COPD	78	56 (27.7)	<0.0001
Patients without COPD	416	137 (12.9)	
Pneumonia or a grade ≥3 infection 1 year before starting ibrutinib presentabsent	118 376	84 (32.3) 109 (10.9)	<0.0001
Patients on TMP-SMX prophylaxis	365	134 (14.4)	0.21
Patients not on TMP-SMX prophylaxis	129	59 (17.6)	
Patients with IgG levels ≤ 400 mg/dL	99	56 (22.1)	0.002
Patients with IgG levels > 400 mg/dL	373	129 (13.2)	
Neutrophil count >1000 ×10^9^/L	416	156 (14.0)	0.001
Neutrophil count ≤1000 × 10^9^/L	49	28 (28.6)	
Rai stage 0–II	234	71 (10.7)	<0.0001
Rai stage III–IV	260	122 (20.2)	
Del17p and/or *Tp*53 mutation present	228	94 (16.5)	0.29
Del17p and *Tp*53 mutation absent	250	92 (14.1)	
IGHV unmutated	319	126 (15.3)	0.98
IGHV mutated	115	46 (15.2)	
Prior treatments 0	89	20 (8.8)	0.003
Prior treatments ≥ 1	405	173 (16.7)	
Prior treatments ≤ 1	258	69 (10.6)	<0.0001
Prior treatments ≥ 2	236	124 (20.2)	
Front-line ibrutinib	65	16 (10.5)	0.23
Front-line ibrutinib+rituximab	24	4 (5.3)	
Patients with concomitant steroids	83	45 (23.2)	0.003
Patients without concomitant steroids	411	148 (13.7)	

Abbreviations: IR, incidence rate; CIRS, Cumulative Illness Rating Scale; CrCL, creatinine clearance; COPD, chronic obstructive pulmonary disease; TMP-SMX, trimethoprim-sulfamethoxazole; IGHV, immunoglobulin heavy chain variable region genes; HBV, Hepatitis B.

**Table 4:** Impact of baseline factors on the time to pneumonia or grade ≥3 infectious events in patients treated with ibrutinib ± rituximab: univariate and multivariate analysis.

	Univariate Analysis HR (95%CI)	Multivariate Analysis HR (95%CI)	Bootstrap Validated Coefficients HR (95% CI)
Pneumonia or grade ≥3 infections 1 year before starting ibrutinib (yes vs. no)	3.09 (2.24–4.27)	2.72 (1.94–3.81)	2.72 (1.93–3.91)
Chronic obstructive pulmonary disease (yes vs. no)	2.23 (1.55–3.21)	1.49 (1.01–2.19)	1.49 (1.01–2.22)
Number of prior treatments (≤1 vs. ≥2)	1.88 (1.36–2.59)	1.67 (1.20–2.33)	1.67 (1.21–2.37)
Number of prior treatments (0 vs. ≥1)	1.99 (1.20–3.29)	NS	
Neutrophil count (≤1000 vs. >1000 × 10^9^/L)	1.83 (1.16–2.88)	NS	
IgG levels (<400 vs. >400 mg/dL)	1.71 (1.20–2.43)	NS	
Steroids (yes vs. no)	1.60 (1.09–2.34)	NS	
RAI stage (III–IV vs. 0–I–II)	1.48 (1.08–2.05)	NS	

**Table 5:** Scoring system to assess the risk of infections in CLL patients treated with ibrutinib ± rituximab.

Variables	Risk Points		
Pneumonia or gr ≥3 infections 1 year before starting ibrutinib	2		
Prior treatments ≥ 2	1		
COPD ^(1)^	1		
**Risk** **Group**	**Risk Score**	**% Patients** **(*n*)**	**% Patients with** **Infections** **(*n*)**
Low	0–1	71.2 (352)	23.0 (81)
Intermediate	2	13.6 (67)	41.8 (28)
High	≥3	15.2 (75)	62.7 (47)

^(1)^ COPD, chronic obstructive pulmonary disease.

## Data Availability

Data may be made available request for consideration to the study corresponding author and establishment of the data transfer agreement.

## Supplementary Materials

The following are available online at https://www.mdpi.com/article/10.3390/cancers13133240/s1, Table S1. Infections recorded in patients treated with ibrutinib ± rituximab, Table S2. Events leading to permanent treatment discontinuation in 2% of patients treated with ibrutinib ± rituximab, Table S3. Cause of death in patients treated with ibrutinib ± rituximab.

## References

[1] 2021 Report of the Surveillance, Epidemiology, and End Results (SEER) Program. https://seer.cancer.gov/accessedon.

[2] International CLL-IPI Working Group (2016). An international prognostic index for patients with chronic lymphocytic leukaemia (CLL-IPI): A meta-analysis of individual patient data. Lancet Oncol..

[3] Burger J.A., Barr P.M., Robak T., Owen C., Ghia P., Tedeschi A., Bairey O., Hillmen P., Coutre S.E., Devereux S. (2020). Long-term efficacy and safety of first-line ibrutinib treatment for patients with CLL/SLL: 5 years of follow-up from the phase 3 RESONATE-2 study. Leukemia.

[4] Woyach J.A., Ruppert A.S., Heerema N.A., Zhao W., Booth A.M., Ding W., Bartlett N.L., Brander D.M., Barr P.M., Rogers K.A. (2018). Ibrutinib Regimens versus Chemoimmunotherapy in Older Patients with Untreated CLL. N. Engl. J. Med..

[5] Moreno C., Greil R., Demirkan F., Tedeschi A., Anz B., Larratt L., Simkovic M., Samoilova O., Novak J., Ben-Yehuda D. (2019). Ibrutinib plus obinutuzumab versus chlorambucil plus obinutuzumab in first-line treatment of chronic lymphocytic leukemia (iLLUMINATE): A multicentre, randomized, open-label, phase 3 trial. Lancet Oncol..

[6] Shanafelt T.D., Wang X.V., Kay N.E., Hanson C.A., O’Brien S., Barrientos J., Jelinek D.F., Braggio E., Leis J.F., Zhang C.C. (2019). Ibrutinib-Rituximab or Chemoimmunotherapy for Chronic Lymphocytic Leukemia. N. Engl. J. Med..

[7] Jain P., Keating M., Wierda W., Estrov Z., Ferrajoli A., Jain N., George B., James D., Kantarjian H., Burger J. (2015). Outcomes of patients with chronic lymphocytic leukemia after discontinuing ibrutinib. Blood.

[8] Maddocks K.J., Ruppert A.S., Lozanski G., Heerema N.A., Zhao W., Abruzzo L.V., Lozanski A., Davis M., Gordon A.L., Smith L.L. (2015). Etiology of Ibrutinib Therapy Discontinuation and Outcomes in Patients with Chronic Lymphocytic Leukemia. JAMA Oncol..

[9] Mato A.R., Nabhan C., Thompson M.C., Lamanna N., Brander D.M., Hill B., Howlett C., Skarbnik A., Cheson B.D., Zent C. (2018). Toxicities and outcomes of 616 ibrutinib-treated patients in the United States: A real-world analysis. Haematologica.

[10] O’Brien S.M., Byrd J.C., Hillmen P., Coutre S., Brown J.R., Barr P.M., Barrientos J.C., Devereux S., Robak T., Reddy N.M. (2019). Outcomes with ibrutinib by line of therapy and post-ibrutinib discontinuation in patients with chronic lymphocytic leukemia: Phase 3 analysis. Am. J. Hematol..

[11] Parikh S.A., Achenbach S.J., Call T.G., Rabe K.G., Ding W., Leis J.F., Kenderian S.S., Chanan-Khan A.A., Koehler A.B., Schwager S.M. (2020). The impact of dose modification and temporary interruption of ibrutinib on outcomes of chronic lymphocytic leukemia patients in routine clinical practice. Cancer Med..

[12] Brown J.R. (2018). How I treat CLL patients with ibrutinib. Blood.

[13] Gribben J.G., Bosch F., Cymbalista F., Geisler C.H., Ghia P., Hillmen P., Moreno C., Stilgenbauer S. (2018). Optimising outcomes for patients with chronic lymphocytic leukaemia on ibrutinib therapy: European recommendations for clinical practice. Br. J. Haematol..

[14] Stephens D.M., Byrd J.C. (2019). How I manage ibrutinib intolerance and complications in patients with chronic lymphocytic leukemia. Blood.

[15] Lasica M., Tam C.S. (2020). Management of Ibrutinib Toxicities: A Practical Guide. Curr. Hematol. Malign Rep..

[16] Morrison V.A. (2014). Infections in Patients with Leukemia and Lymphoma. Cancer Treat. Res..

[17] Peri A.M., Rossio R., Tafuri F., Benzecry V., Grancini A., Reda G., Bandera A., Peyvandi F. (2019). Atypical primary cutaneous cryptococcosis during ibrutinib therapy for chronic lymphocytic leukemia. Ann. Hematol..

[18] Koehler A.B., Vijayvargiya P., Ding W. (2019). Probable Invasive Pulmonary Cryptococcosis and Possible Cryptococcal Empyema in CLL Treated with Frontline Ibrutinib. Mayo Clin. Proc..

[19] Giridhar K.V., Shanafelt T., Tosh P.K., Parikh S.A., Call T.G. (2016). Disseminated herpes zoster in chronic lymphocytic leukemia (CLL) patients treated with B-cell receptor pathway inhibitors. Leuk. Lymphoma.

[20] Lutz M., Schulze A.B., Rebber E., Wiebe S., Zoubi T., Grauer O.M., Keßler T., Kerkhoff A., Lenz G., Berdel W.E. (2017). Progressive Multifocal Leukoencephalopathy after Ibrutinib Therapy for Chronic Lymphocytic Leukemia. Cancer Res. Treat..

[21] Raisch D.W., Rafifi J.A., Chen C., Bennett C.L. (2016). Detection of cases of progressive multifocal leukoencephalopathy associated with new biologicals and targeted cancer therapies from the FDA’s adverse event reporting system. Expert. Opin. Drug Saf..

[22] Wang S.-Y., Ebert T., Jaekel N., Schubert S., Niederwieser D., Al-Ali H.K. (2015). Miliary tuberculosis after initiation of ibrutinib in chronic lymphocytic leukemia. Ann. Hematol..

[23] Rogers K.A., Mousa L., Zhao Q., Bhat S.A., Byrd J.C., El Boghdadly Z., Guerrero T., Levine L.B., Lucas F., Shindiapina P. (2019). Incidence of opportunistic infections during ibrutinib treatment for B-cell malignancies. Leukemia.

[24] Ghez D., Calleja A., Protin C., Baron M., LeDoux M.-P., Damaj G., Dupont M., Dreyfus B., Ferrant E., Herbaux C. (2018). Early-onset invasive aspergillosis and other fungal infections in patients treated with ibrutinib. Blood.

[25] Ruchlemer R., Ben-Ami R., Bar-Meir M., Brown J.R., Malphettes M., Mous R., Tonino S.H., Soussain C., Barzic N., Messina J.A. (2019). Ibrutinib-associated invasive fungal diseases in patients with chronic lymphocytic leukaemia and non-Hodgkin lymphoma: An observational study. Mycoses.

[26] Varughese T., Taur Y., Cohen N., Palomba M.L., Seo S.K., Hohl T.M., Redelman-Sidi G. (2018). Serious Infections in Patients Receiving Ibrutinib for Treatment of Lymphoid Cancer. Clin. Infect. Dis..

[27] Zarakas M.A., Desai J.V., Chamilos G., Lionakis M.S. (2019). Fungal Infections with Ibrutinib and Other Small-Molecule Kinase Inhibitors. Curr. Fungal Infect. Rep..

[28] Ahn I.E., Jerussi T., Farooqui M., Tian X., Wiestner A., Gea-Banacloche J. (2016). Atypical *Pneumocystis jirovecii* pneumonia in previously untreated patients with CLL on single-agent ibrutinib. Blood.

[29] Ryan C.E., Cheng M.P., Issa N.C., Brown J.R., Davids M.S. (2020). *Pneumocystis jirovecii* pneumonia and institutional prophylaxis practices in CLL patients treated with BTK inhibitors. Blood Adv..

[30] Herishanu Y., Katchman H., Polliack A. (2017). Severe hepatitis B virus reactivation related to ibrutinib monotherapy. Ann. Hematol..

[31] Hammond S., Chen K., Pandit A., Davids M.S., Issa N.C., Marty F.M. (2018). Risk of hepatitis B virus reactivation in patients treated with ibrutinib. Blood.

[32] Williams A.M., Baran A.M., Meacham P.J., Feldman M.M., Valencia H.E., Newsom-Stewart C., Gupta N., Janelsins M.C., Barr P.M., Zent C.S. (2018). Analysis of the risk of infection in patients with chronic lymphocytic leukemia in the era of novel therapies. Leuk. Lymphoma.

[33] Tillman B.F., Pauff J.M., Satyanarayana G., Talbott M., Warner J.L. (2018). Systematic review of infectious events with the Bruton tyrosine kinase inhibitor ibrutinib in the treatment of hematologic malignancies. Eur. J. Haematol..

[34] Coutre S.E., Byrd J.C., Hillmen P., Barrientos J.C., Barr P.M., Devereux S., Robak T., Kipps T.J., Schuh A., Moreno C. (2019). Long-term safety of single-agent ibrutinib in patients with chronic lymphocytic leukemia in 3 pivotal studies. Blood Adv..

[35] Ball S., Das A., Vutthikraivit W., Edwards P.J., Hardwicke F., Short N.J., Borthakur G., Maiti A. (2020). Risk of Infection Associated with Ibrutinib in Patients With B-Cell Malignancies: A Systematic Review and Meta-analysis of Randomized Controlled Trials. Clin. Lymphoma Myeloma Leuk..

[36] Kohrt H.E., Sagiv-Barfi I., Rafiq S., Herman S.E.M., Butchar J.P., Cheney C., Zhang X., Buggy J.J., Muthusamy N., Levy R. (2014). Ibrutinib antagonizes rituximab-dependent NK cell-mediated cytotoxicity. Blood.

[37] Borge M., Almejún M.B., Podaza E., Colado A., Grecco H.F., Cabrejo M., Bezares R.F., Giordano M., Gamberale R. (2015). Ibrutinib impairs the phagocytosis of rituximab-coated leukemic cells from chronic lymphocytic leukemia patients by human macrophages. Haematologica.

[38] Herbst S., Shah A., Moya M.M., Marzola V., Jensen B., Reed A., Birrell M.A., Saijo S., Mostowy S., Shaunak S. (2015). Phagocytosis-dependent activation of a TLR 9- BTK -calcineurin- NFAT pathway co-ordinates innate immunity to *Aspergillus* fumigatus. EMBO Mol. Med..

[39] Bercusson A., Colley T., Shah A., Warris A., Armstrong-James D. (2018). Ibrutinib blocks Btk-dependent NF-ĸB and NFAT responses in human macrophages during *Aspergillus fumigatus* phagocytosis. Blood.

[40] Woyach J.A. (2018). Ibrutinib and Aspergillus: A Btk-targeted risk. Blood.

[41] Maffei R., Maccaferri M., Arletti L., Fiorcari S., Benatti S., Potenza L., Luppi M., Marasca R. (2020). Immunomodulatory effect of ibrutinib: Reducing the barrier against fungal infections. Blood Rev..

[42] Blez D., Blaize M., Soussain C., Boissonnas A., Meghraoui-Kheddar A., Menezes N., Portalier A., Combadière C., Leblond V., Ghez D. (2019). Ibrutinib induces multiple functional defects in the neutrophil response against Aspergillus fumigatus. Haematologica.

[43] Hallek M., Cheson B.D., Catovsky D., Caligaris-Cappio F., Dighiero G., Döhner H., Hillmen P., Keating M., Montserrat E., Chiorazzi N. (2018). iwCLL guidelines for diagnosis, indications for treatment, response assessment, and supportive management of CLL. Blood.

[44] Ward M.D., Ahlquist J.S. (2018). Maximum Likelihood for Social Science: Strategies for Analysis.

[45] Byrd J.C., Hillmen P., O’Brien S., Barrientos J.C., Reddy N.M., Coutre S., Tam C.S., Mulligan S.P., Jaeger U., Barr P.M. (2019). Long-term follow-up of the RESONATE phase 3 trial of ibrutinib vs ofatumumab. Blood.

[46] Byrd J.C., Furman R.R., Coutre S.E., Flinn I.W., Burger J.A., Blum K.A., Sharman J.P., Wierda W., Zhao W., Heerema N.A. (2020). Ibrutinib Treatment for First-Line and Relapsed/Refractory Chronic Lymphocytic Leukemia: Final Analysis of the Pivotal Phase Ib/II PCYC-1102 Study. Clin. Cancer Res..

[47] Treon S.P., Castillo J.J., Skarbnik A.P., Soumerai J.D., Ghobrial I.M., Guerrera M.L., Meid K.E., Yang G. (2020). The BTK inhibitor ibrutinib may protect against pulmonary injury in COVID-19–infected patients. Blood.

[48] Cuneo A., Scarfò L., Reda G., Varettoni M., Quaglia F.M., Marchetti M., de Paoli L., Re F., Pietrasanta D., Rigolin G.M. (2020). Chronic lymphocytic leukemia management in Italy during the COVID-19 pandemic: A Campus CLL report. Blood.

[49] Reda G., Noto A., Cassin R., Zamprogna G., Borella C., Scarfò L., Farina L., Molteni A., Ghia P., Tedeschi A. (2020). Reply to “CLL and COVID-19 at the Hospital Clinic of Barcelona: An interim report” Analysis of six hematological centers in Lombardy. Leukemia.

[50] Baumann T., Delgado J., Montserrat E. (2020). CLL and COVID-19 at the Hospital Clinic of Barcelona: An interim report. Leukemia.

[51] Scarfò L., Chatzikonstantinou T., Rigolin G.M., Quaresmini G., Motta M., Vitale C., Garcia-Marco J.A., Hernández-Rivas J. (2020). Ángel; Mirás, F.; Baile, M.; et al. COVID-19 severity and mortality in patients with chronic lymphocytic leukemia: A joint study by ERIC, the European Research Initiative on CLL, and CLL Campus. Leukemia.

[52] Mato A.R., Roeker L.E., Lamanna N., Allan J.N., Leslie L., Pagel J.M., Patel K., Osterborg A., Wojenski D., Kamdar M. (2020). Outcomes of COVID-19 in patients with CLL: A multicenter international experience. Blood.

[53] Maschmeyer G., de Greef J., Mellinghoff S.C., Nosari A., Thiebaut-Bertrand A., Bergeron A., Franquet T., Blijlevens N.M.A., Maertens J.A. (2019). Infections associated with immunotherapeutic and molecular targeted agents in hematology and oncology. A position paper by the European Conference on Infections in Leukemia (ECIL). Leukemia.

[54] Byrd J.C., Hillmen P., Ghia P., Kater A.P., Chanan-Khan A.A.A., Furman R.R., O’Brien S.M., Yenerel M.N., Illés Á., Kay N.E. (2021). First results of a head-to-head trial of acalabrutinib versus ibrutinib in previously treated chronic lymphocytic leukemia. J. Clin. Oncol..

[55] Tam C.S., Trotman J., Opat S., Burger J.A., Cull G., Gottlieb D., Harrup R., Johnston P.B., Marlton P., Munoz J. (2019). Phase 1 study of the selective BTK inhibitor zanubrutinib in B-cell malignancies and safety and efficacy evaluation in CLL. Blood.

[56] Hofland T., de Weerdt I., Ter Burg H., de Boer R., Tannheimer S., Tonino S.H., Kater A.P., Eldering E. (2019). Dissection of the Effects of JAK and BTK Inhibitors on the Functionality of Healthy and Malignant Lymphocytes. J. Immunol..

