# Supplementary material for: Prognostic Impact and Risk Factors of Infections in Patients with Chronic Lymphocytic Leukemia Treated with Ibrutinib
Source: Cancers (Basel). 2021 Jun 29;13(13):3240. doi: 10.3390/cancers13133240 (PMC8269042; doi:10.3390/cancers13133240)
Supplement: Supplementary file 1 [file cancers-13-03240-s001.zip › cancers-1242967-supplementary.pdf]

Supplementary Material

Table S1. Infections recorded in patients treated with ibrutinib ± rituximab.

Total number of infectious events [number of lethal infections]	156 (29)
Pneumonia [(1)]	126 (17)
Grade ≥ 3 non opportunistic infections	40 (3)
Sepsis[(2)]	7 (2)
Other non opportunistic infections[(3)]	26 (0)
Viral infections[(4)]	7 (1)
Grade ≥ 3 opportunistic infections OI	25 (9)
Fungal infections	14 (7)
• *Aspergillus*[(5)]	11 (6)
• *Criptococcus*[(6)]	2 (1)
• *Pneumocystis jirovecii (PJ)*	1 (0)
Mycobacterium tuberculosis	2 (0)
CMV infection[(7)]	3 (1)
Disseminated HVZ	5 (0)
PML	1(1)

Abbreviations: IR, incidence rate; IBR±R, ibrutinib+rituximab; CMV, cytomegalovirus, HVZ, herpes varicella zoster; PML, Progressive multifocal leukoencephalopathy. [(1)] Pneumonia etiology: pneumococcus, 3 cases; pseudomonas, 2; cytrobacter Kyusei, 1; actinomyces, 1; not specified Gam+ agent, 1; not specified Gam-, agent, 1; not specified,117 . [(2)]Sepsis: *E. Coli*, 3 cases; *Klebsiella*, 1; *Staphylococcus aureus*, 1; not specified, 2.[(3)]Other infections: soft tissues infections, 5; gastroenteric, 6; urogenital, 7; upper respiratory tract infections, 8. [(4)]Viral infections: *H1N1* influenza, 1 case; reactivation of HBV hepatitis, 3 HBc positive patients; Covid-19 pneumonia, 2; EBV infection, 1. [(5)] Aspergillus infections: pneumonia, 7 cases; sepsis,3; pneumonia and cerebral involvement, 3, spondilodiscitis, 1.[(6)] Criptococcus infection: pneumonia,1 case; meningitis, 1.[(7)] CMV infection: CMV gastroenteritis, 1 case; CMV interstitial pneumonia, 2.

Table S2. Events leading to permanent treatment discontinuation in 2% of patients treated with ibrutinib ± rituximab.

	***n* = 494 (100%)**
No patients with permanent treatment discontinuation	236 (48)
Disease progression	80 (16)
Infections	43 (9)
Richter syndrome	27 (5)
Atrial fibrillation	23 (5)
Second malignancies	18 (4)

Table S3. Cause of death in patients treated with ibrutinib ± rituximab.

	***n* = 494 (%)**
No of deaths	95 (19)
Infection	29 (6)
Disease progression	30 (6)
Richter syndrome	24 (5)
Second malignancies	12 (2)
